# Tending The Garden: What organic youth peer support can teach us about building systems that care

**DOI:** 10.1371/journal.pmen.0000678

**Published:** 2026-10-21

**Authors:** Ernesto Isaac Lara, Rhianen Callahan, Leana Cortez, Kharylle Rosario, Anna Zick, Rocio Sanchez, Aishwarya Mitra, Megha Datta, Gabriella Imai

**Affiliations:** 1 Department of Global Health and Social Medicine, Harvard Medical School, Boston, Massachusetts, United States of America; 2 Department of Health Policy and Management, UCLA Fielding School of Public Health, Los Angeles, California, United States of America; 3 Youth MOVE National, United States of America; 4 Independent, United States of America; PLOS: Public Library of Science, UNITED KINGDOM OF GREAT BRITAIN AND NORTHERN IRELAND

## Abstract

Youth peer support (YPS) is an evidence-based model of care in which young people with lived experience (YPWLE) provide recovery-oriented support to peers navigating similar challenges. On many college campuses this work already happens informally, without training, compensation, or institutional recognition. This narrative essay examines The Garden, a unit of a student mentor-mentee program at the University of California, San Diego, that organically evolved into de facto YPS, providing students mutual aid spanning acute crisis, identity formation, and navigation of the hidden curriculum of higher education. Drawing on first-person accounts, we show that The Garden often operated in place of institutional mental health and advising systems, providing immediate, person-centered support grounded in shared lived experience and human rights. A competency mapping across these accounts demonstrates that The Garden consistently reflected practices the YPS literature identifies as central and effective. We argue that higher education largely lacks both the infrastructure and the institutional power reflexivity to recognize, train, and sustain this labor, manufacturing an invisible and inequitable burden for students. We call on higher education institutions to meaningfully invest in YPS infrastructure, centering youth leadership and community ownership. When institutions seek to scale YPS without ceding power they risk co-optation, stripping student spaces of the autonomy and trust that make them effective and accessible. Drawing on youth participation scholarship, we distinguish recognition from control and propose recommendations across policy, funding, programming, campus climate, and research to build YPS infrastructure that reflects students’ lived realities.

## Preface

This narrative essay is written collectively in the first person, by young people with lived experience (YPWLE) of mental health challenges, marginalization, and of both receiving and providing peer support across a range of crises (see S1 Text).

This essay was developed through a collaborative co-authorship process, in which all co-authors voluntarily joined the project since its inception. A group meeting was initially conducted to conceptualize the piece, including its goals, scope, boundaries, and consent process. Each author wrote their own vignette and retained full control over what they chose to share; no disclosures were required or prompted. Instances where authors are referenced by another were reviewed and approved by the individuals named. All co-authors reviewed and approved the full manuscript prior to submission and retained the right to withdraw or modify their contributions at any point. Given the mentorship relationship between EIL and most co-authors, the voluntary nature of participation was communication explicitly and repeatedly, both verbally and in writing. This essay constitutes collaborative co-authorship, rather than human subjects research, thus formal ethics review was not sought.

This medium was intentionally chosen as a form of epistemic justice, resting on the premise that YPWLE are credible narrators of their own care, not simply research subjects of it. In this piece, lived experience is treated as primary evidence, with scholarship primarily serving as a means of contextualizing our experiences within broader documented patterns examined by the field, as well as identifying instances where our accounts exceed what existing frameworks have captured. This essay is a reflection of our lived realities, demonstrating the nuances of young people’s experiences of crisis, and the ways young people often become de facto youth peer supporters.

## Introduction

I (EIL) often joke that I graduated college as the parent of fifteen children, in reference to the mentees I supported as an Undergraduate Student Mentor at the University of California, San Diego (UCSD). Although my role, on paper, was designed to focus on academic guidance, campus navigation, and stress management, it quickly evolved into something more. My mentees were navigating complex mental health challenges, trauma, marginalization, identity crises, and institutional failures in a system where traditional models of support were often inaccessible, and culturally inadequate.

Despite the availability of various mental health and affinity-specific resources at UCSD, more often than not students received generalized services that failed to recognize the complexity of students’ lived experiences, including extensive wait times for counseling services, burdensome disability accommodations processes, and affinity spaces that were too often “one size fits all” [1,2]. The challenges we faced are reflective of broader barriers pervading higher education, where limited funding, a strained mental health workforce, and deepening distrust in health systems have converged into an increasingly challenging and under-resourced mental health ecosystem [3,4]. These campus-level dynamics echo the challenges faced in global contexts arguing for an intersectional, youth-led, rights-based response to address the youth mental health crisis [5,6].

In response to the barriers we faced, my mentees and I turned to one another, creating The Garden: an informal peer support network grounded in mutual aid, recovery, and activism. The Garden originated in our student organization’s mentor-mentee or “big-little” system, a peer mentorship structure common across student organizations in American higher education that pairs newer students with experienced ones, creating “family units” built on guidance, belonging, and support. Research consistently links peer mentorship systems to improved academic outcomes, stronger sense of belonging, and meaningful improvements to overall mental wellbeing, especially for students that are first-generation college graduates and from historically marginalized communities [7–9]. Although we began as an assigned peer mentorship program, our relationships organically evolved into something we, at the time, did not have the language to describe: youth peer support (YPS).

The Garden was not a professionalized YPS program, but it was by substantive definition YPS, operating within an institution that had every reason to invest in it and consistently resisted doing so. What distinguished The Garden from informal friendship was its origin in an explicitly asymmetric mentoring structure, the deliberate mobilization of shared lived experience as the basis for support, recognized roles within a student organization, and intentionality. Members of The Garden were not simply friends who happened to help one another, but peers who mobilized their own experiences in response to recognized needs. As mentors and mentees, we were not trained, supervised, nor provided institutional recognition to deliver YPS services. However, the support infrastructure we built reflected the core values the peer movement was founded on: mutuality, shared lived experience, recovery, and the creation of alternative, community-owned systems of care [10,11].

YPS, in contemporary practice, refers to a formalized model of mental healthcare in which YPWLE are intentionally trained, supervised, and compensated to provide recovery-oriented support across the mental health ecosystem [12]. A growing evidence base demonstrates peer-based interventions’ ability to reduce suicide attempts among school-aged students and improve belonging, coping, and mental health outcomes in higher education settings; this includes via upstream peer-leader programs such as Sources of Strength [9,13–15]. For young people from marginalized communities specifically, access to YPS is associated with significantly increased mental health service utilization and reduced racial disparities in access [16]. Despite this evidence, and global calls to center YPWLE in mental health solutions, adoption across higher education remains uneven: while 75% of US campuses enrolled in the Healthy Minds Network offer at least one peer support program, high-touch models that intentionally position students as direct providers of mental health support remain the least adopted [17].

The Garden is likely not unique. Across higher education settings, students frequently run and participate in affinity-based organizations, clubs, fraternities/sororities, and additional forms of student life that regularly employ a peer mentorship system. Young people are building their own systems of care, either through formal peer-based mental health interventions or through organically fostered YPS, filling in the spaces institutions have left unfilled. Young people are actively serving as youth peer supporters, providing crisis prevention and intervention; navigating complex academic bureaucracies; and doing the work without the infrastructure. When institutions do acknowledge this labor, the response is frequently cautious, preceded with concerns of liability, assumptions of incompetence, and implicit ageism that positions young people as only capable of being recipients rather than providers of care. This dynamic results in a structural paradox in which universities simultaneously benefit from and depend on informal forms of YPS, yet refuse to trust or invest in it.

Universities are the primary beneficiaries, and too often the primary obstacles, of student-created YPS systems. Collectively, we argue that youth-created systems of care must be recognized, resourced, and led by the students who built them. In the sections that follow, we examine how YPS manifested across mentor-mentee relationships, spanning mental health crises, identity formation, and systems navigation.

## Crisis, care, and institutional limits

While UCSD and many universities share a similar definition of crisis, recent literature challenges that framing, calling for an intersectional expansion that accounts for the diverse ways students face, experience, and describe their own crises [18,19]. UCSD’s institutional response was primarily reactionary, narrowing crisis to “acute psychiatric distress,” without remedying the institutional drivers that promote harm. By narrowing the definition of a crisis, higher education administrations are enabled to govern who receives care, whose situation is deemed “severe enough,” and leverage “safety concerns” as a means of control over students [20,21].

Crisis, as our four accounts demonstrate below, arrived across a spectrum of mental, physical, and material crises, each demanding personalized support that the institution’s generalist model was unable to provide:

*“As a student leader, I (EIL) had publicly advocated for expanding access to mental health services and personally knew UCSD’s Director of Counseling and Psychological Services (CAPS). Despite having knowledge and relationships within the very system designed to help, none of it was enough for me to seek care. During my sophomore year, at the onset of the COVID-19 pandemic, I collapsed into severe depression and suicidality, struggling with chronic exhaustion, debilitating sadness, and seemingly endless spiraling. Despite these challenges, I still did not reach out to CAPS. Cultural and internalized stigma had convinced me that other students’ crises were more urgent than my own, and that I had not yet ‘earned the right’ to seek care.*

*What allowed me to survive, even at my lowest points, were my mentees. They brought me food when I had no energy to get out of bed and eat. They walked with me for hours through campus in the middle of the night when the depression was at its worst. And they sat with me while I processed the same grief, again and again, without ever making me feel like a burden. My mentees became my safety net, although they were not intended to.”*

EIL’s account embodies what research increasingly demonstrates: that help-seeking among college students is shaped less by resource awareness than by internalized stigma, self-perceived severity, and distrust of institutional systems [22,23]. For students of color, first-generation college students, and LGBTQ+ students, these barriers are compounded by historical harms within mental health systems and the persistent absence of culturally responsive care [24,25]. Peer relationships are uniquely positioned to bridge this gap precisely because they operate outside institutional power dynamics, without waitlists, severity thresholds, or any requirement to justify one’s pain [26,27]. Crisis, however, does not always arrive as psychiatric distress, as the following account by AZ reveals how institutional indifference worsened physical crisis:

*“In 2022, I (AZ) tore my meniscus and ACL in a skiing accident. Almost immediately, anxieties set in: I was graduating in June, living off-campus, working three part-time jobs, caring for an elderly pet and my family’s home, and attending school full time. I quickly began to struggle physically, mentally, and academically. I was already wary of CAPS after negative experiences, and hesitated to contact them.*

*What followed showed me where institutional support ends and community begins. Peers brought groceries, fed my cats, and sat with me while I called doctors and navigated insurance. These ‘simple’ acts of support allowed me to finish the quarter in good standing.*

*When classes returned in-person, getting around UCSD’s sprawling campus became a new barrier. I reached out to the Office for Students with Disabilities (OSD), but they were non‑responsive. Professors referred me back to OSD, trapping me in a loop that left me to get through the quarter alone.*

*My community refused to let that happen. I never walked to class by myself; someone always picked me up, carried my bag, and got me to the door. When surgery was scheduled four weeks before graduation, The Garden helped me secure accommodations. My degree bears the UC seal, but it was my community that carried me across the stage.”*

Similar to AZ, RC faced their own institutional ableist barriers navigating UCSD’s physical and accommodations landscape:

*“In my first quarter of my first year, I (RC) fell off my bottom bunk while sleeping, hitting my neck on a trash can rim and my head against the wooden desk. I lived in a cramped triple room meant for a double. It was two weeks later, when I started waking up nauseous, sensitive to sound and light, and nearly fainted walking to class. When my roommate took me to the emergency room. The doctor’s first question was how long I’d had a concussion.*

*Two weeks untreated. I had to stop all coursework immediately. My doctor referred me to OSD and my college’s academic affairs team. My college’s team was the difference between recovery and long-term damage: they checked in, helped me withdraw from classes, connected me with CAPS, and moved me from the triple into a single so I could actually rest. They handled what a student with a brain injury cannot handle alone.*

*OSD was the opposite: They insisted I come in-person, without directions; denied accommodations my doctor requested; and required me to hunt down professors for signatures while concussed. Asking a disoriented student to navigate a campus alone in the elements they should have been avoiding to heal is the kind of abandonment OSD has become known for. Years of student campaigns and administration negotiations produced little progress. A Disability Resource Center finally opened this past year. The students who needed it while I was there deserved better than to become the evidence that eventually moved administration to act.”*

OSD’s mandate is to ensure equal educational access for students with disabilities, yet its accommodation processes requiring students to face heavy scrutiny, complete lengthy documentation, and essentially justify their needs, exacerbate the same inaccessibility they are designed to prevent [28,29]. RC and AZ’s accounts exemplify the ways in which bureaucratic complexity and the burden of self-advocacy serve as primary barriers to seeking and receiving academic accommodations [30–32]. RC’s positive experience with their academic affairs team reveals humane support was institutionally possible, proving OSD’s failure was not an inevitability. The Garden’s actions embodied interdependence and collective care, grounded in disability justice principles, the same foundations that helped shape the peer support movement itself [33,34]. While both AZ and RC were able to receive support, what The Garden provided was evidence that the institution’s failure had already been absorbed by community, invisibly and unrecognized. Just as peers within The Garden bridged the gap left by inaccessible accommodation processes, they also became the first and often only line of response when institutional failures threatened our most basic needs:

*“In 2018, I (MD) moved from Hong Kong to UCSD. Being a 16-hour flight from home and trying to ‘adult’ for the first time in a foreign cultural landscape was one of my most daunting experiences. Adding to this alienation, being a third-culture kid – someone who grew up immersed in cultures different from my parents’ own, without fully belonging to any single one – meant I didn’t fit into any nationality-specific student group on campus. My first year was incredibly isolating; I only focused on surviving day to day. That changed when I met EIL, GI, and The Garden. With them, I finally felt safe and belonging, being able to ask ‘stupid’ questions and thrive in college.*

*The Garden taught me that true peer support does not just provide emotional support, but also acts as a critical safety net for basic needs. In March 2020, international students were urged to return home immediately due to potential border closures. I had six hours to pack my dorm, find storage, and get to the airport; without a car or help, it seemed impossible, until GI arrived. She packed my room, drove us to storage, and rushed me to my flight.”*

Across our lived experiences, structural and interpersonal challenges amplified our struggles in ways that mirror broader trends in higher education. Scholars have shown that the college years are developmentally sensitive and highly transitional with academic pressure, financial strain, social isolation, and relationship stress accumulating alongside pre-existing mental health challenges [35–37]. In this context, everyday and proximal stressors can generate substantial distress and impairment, regardless whether a student has a formal diagnosis or received mental health services prior [36,37].

UCSD did not build The Garden, but it benefited from the crisis-response system we created: a system that was supportive, and unsustainable. The literature consistently identifies the same elements of peer-delivered crisis support that shaped The Garden’s response, including rapid mobilization, wraparound care, and sustained presence [14,18,26]. The support we provided (e.g., financial aid, housing, transportation) exceeds the boundaries of professionalized YPS roles, whose scope is limited to emotional and recovery-oriented support to precisely avoid this kind of entanglement that could pose a conflict of interest [15]. Our collective accounts reveal that The Garden was operating in place of a functioning mental healthcare system.

When student-created, youth-led models of care are intentionally resourced and embeded within college mental health systems, they reduce stigma, increase mental health knowledge and helping behaviors, and produce distinct levels of care that formal services cannot replicate [9,38]. Higher education does not need to reinvent YPS, it must enact a redistribution of trust and resources toward the YPS students are already providing one another.

## Identity and belonging

The developmental volatility often experienced by YPWLE in higher education extends beyond crisis into identity. As young people construct their sense of self, peer relationships, campus climate, and safe and affirming spaces become key contributors to confidence and wellbeing [39,40]. Students from marginalized communities encounter this landscape as one structured around their exclusion. Vulnerable to microaggressions, discrimination, and hostile campus climates, students of color face elevated academic and mental health challenges and undermined senses of belonging [41–44].

Many universities, including UCSD, have attempted to address these needs through institutionally owned resources: affinity-based resource centers, identity-specific programming, and culturally-informed counseling services. These efforts represent genuine institutional investment and have meaningfully supported some students, but they operate within the political origins of the institution itself. American higher education is not politically neutral, emerging from and materially sustained by the exploitation of enslaved and colonized people [45,46]. Within this history, the University of California system was endowed through land taken from Indigenous nations; UCSD specifically sitting on the unceded territory of the Kumeyaay Nation [47,48]. UCSD’s history is a case study of how discrimination and oppression pervade campus climate; in 2010 a series of anti-Black incidents, known as “Black Winter,” exposed a racist campus climate that the university has still yet to repair [49,50]. The resource centers and affinity-based resources that exist today are the reactionary response of the administration to students’ demands following these very events [51–53].

As queer and racialized students, we navigated our own identities at UCSD, contending with a historically exclusionary institution whose diversity infrastructure, no matter how well-intentioned, remains a product of the structural problem. The four accounts below document what happened when these institutional spaces could not hold that complexity, and what YPS provided in its place:

*“I (KR) immigrated to the United States from the Philippines at 13. As the first in my family to study in the US, I navigated higher education largely alone, attributing my academic success thus far to luck, not ability. The imposter syndrome was exacerbated by the COVID-19 pandemic. Entering UCSD virtually in 2020, I hesitated to get involved in anything since building community without tangibility proved difficult.*

*When restrictions lifted, I met EIL at a tabling event for Associated Students. His encouragement led me to The Garden, a space that promoted my sense of belonging as he and my fellow mentees showed me how to navigate the university’s intricacies and pushed me to strive for leadership roles I had convinced myself I did not deserve.*

*Three years later, completing my MSc in London, I still carry The Garden with me. It is because of them that I now dare to build community in places that discourage it.”*

American higher education is structurally built around cultural fluency and privileges citizenship status, producing challenges for immigrant and international students that compound in ways the institution rarely acknowledge as interconnected [54,55]. Language adjustment, academic unfamiliarity, and the slow erosion of cultural identity under assimilation pressure are each difficult enough in isolation. The international student experience, with the current infrastructure, is particularly hard to support because these barriers arrive simultaneously, alongside visa precarity and geographic distance from the family networks that might otherwise absorb some of the weight [55–57]. Campus programming tends to address these as separate problems with separate solutions, failing to provide the holistic support these students require [56,58]. The Garden provided KR and MD personalized, holistic support, offering shared cultural affinity, practical navigation, and a safe, non-judgmental space to be understood. For others in The Garden, belonging was defined not by navigating a foreign system, but finding a space where they did not need to justify who they are:

*“I (AZ) grew up in a conservative Christian household where being queer was firmly unacceptable. I spent my late teens and early 20s grappling with the extreme Christian beliefs I was raised with while struggling with my own bisexuality. I was 16 when I first kissed a girl, and 22 before I said it out loud to another person.*

*When I transferred to UCSD, I was still trying to hold two identities at once: my faith and my support for the queer people in my life. I sought community in clubs and organizations, but more often than not, I left feeling more alone. For nearly a year, I saw a CAPS counselor, but never once unpacked my queerness with her. I slowly found community in The Garden, finding a space to discuss my identities. The Garden did not need me to have figured anything out. They let me voice beliefs I was unlearning, with patience and without judgement. In those raw, vulnerable spaces I finally felt safe enough to say out loud that I was queer, all while continuing to create a new relationship with religion.”*

AZ describes a critical gap between the designated spaces institutions provide and the nuanced needs students have in order to feel truly safe. For students navigating their gender identity, the institutional response often widens that gap:

*“My (RC) first exposure to the concept of gender identity and expression was during first-year orientation. I admit I felt like a fish out of water, leaning on my orientation leader for support and constantly asking questions. Looking back, it was the first time something deep within me felt seen.*

*College being my first exposure to gender identity did not make the institution’s systems easier to navigate. If I had a dollar for every time an administrator complained about my pronouns making things ‘difficult’ for them, or gave passive-aggressive body language after being corrected, I would have a steady income.*

*The Garden gave me space to experiment with my gender identity privately, and when I was ready, publicly. I am forever grateful for their openness as I became more comfortable with myself, and for their receptiveness to my changing pronouns. They backed me up in administrative spaces. When I faced identity-targeted bullying, they came to my defense in ways I could not repay. They were with me through the entire process of wanting, applying, waiting for, and receiving top surgery — visiting afterward, helping me dress, following me around to open doors when I could not lift anything.”*

Taken together, these accounts underscore a central distinction: it is not enough for universities to simply add YPS into existing systems of care. By maintaining institutional authority over YPS programs, governed by liability and risk management, many of the students who may most benefit from such services may not use them. Students from marginalized communities carry stigma about mental health and, in many cases, collective memories of psychiatric abuse and coercion [24,25]. University branded YPS programs can be perceived as extensions of the same structures that are actively driving their marginalization.

In contrast, students frequently find connection and safety in organizations and support systems that are not owned by the university [59,60]. Affinity-based, student-run organizations and programs, such as the Student Promoted Access Center for Education & Service (SPACES) frequently employ some form of a family or peer mentorship program, functioning as de facto YPS [12,17,61]. These community‑owned networks are approachable precisely because they do not require disclosing stigmatized identities or diagnoses, and because they offer sustained, culturally grounded support on students’ own terms [24,25]. The role of the university, then, is not to replace these ecosystems with their own branded version, but to fund and protect the student-led programs that already exist. Leveraging funding, infrastructure and policy shields, universities can support these efforts without folding them back into the systems they were built to be an alternative to.

## Translating the hidden curriculum

Academia’s infrastructure implicitly presumes that students arrive knowing how to be students: how to find resources, communicate with faculty, navigate institutional bureaucracy, and adapt to college-level expectations. Universities attempt to bridge these gaps through, often meaningful, incoming student orientations, community-specific programs (e.g., first-generation student programming), and student-led events striving to build community [7,62]. However, these programs rarely address the hidden curriculum directly: the unwritten rules and norms institutions consistently reward but don’t teach, leaving students to absorb them by trial and error and/or proximity to someone who holds cultural fluency [63,64].

For students of color, the hidden curriculum often includes the expectation to code-switch, shifting their external presentation to move safely through a system organized around a narrow cultural default [65,66]. Sustained code-switching is cognitively and emotionally taxing, linking to strain on students’ mental health, sense of belonging, and academic engagement [67,68]. In practice, the hidden curriculum functions as a gatekeeping mechanism. Students are not assessed for their academic competence, but for cultural fluency that the university implicitly treats as a baseline [53,69]. Those who can decode and perform it are rewarded with smoother access to opportunities, mentoring, and professional pathways, while those who cannot are more likely to experience poorer academic outcomes and wellbeing and to be framed as “falling behind” [41,70]. Students who arrive without this fluency are not deficient, but are asked to interpret and succeed within a system built around the historical exclusion of their families and communities [45,71].

The three accounts below trace what it looks like when students encounter the hidden curriculum and how YPS became their teachers and translators:

*“I (RS) wasn’t 100% sure I would even show up for UCSD’s move-in day. The weight of moving away and leaving my immigrant parents behind was overwhelming. My first interactions with my peers were discussions about SAT scores, AP classes, and extracurriculars. I immediately knew I didn’t measure up to them at all; I was surrounded by overachievers who didn’t look like me or share my background.*

*Anxiety began to consume me and my imposter syndrome grew only larger by the day. I felt intimidated to approach professors, too uncertain to access campus resources, and convinced that what I was going through was not serious enough for CAPS.*

*I felt it was finally time to make some connections after my first year getting interrupted by the COVID-19 pandemic. When I mustered up the courage to join an organization on campus, Thurgood Marshall College Student Council, I met my support group of mentors (soon turned into best friends) where I leaned on them for empathy, shared lived experiences, and support, at a time when campus resources intimidated me. They helped me drastically with guidance and I carry them with me in every space I enter through life, and without them I would not still be here today.”*

RS’s account demonstrates how easily uncertainty can harden into avoidance when every basic question feels like evidence of not belonging. In an environment that idolizes a certain portrayal of student success, it is quietly communicated that support is for students who already speak “the language” of higher education. LC’s experience traces a similar arc:

*“I (LC) am the first in my family to attend university. At first, I took immense pride in figuring everything out on my own; applications, financial aid, choosing a degree, and imagining my future. That confidence began to falter when I arrived at UCSD. My anxiety spiraled and I rethought my career path while navigating grief and the pressures of school and employment simultaneously.*

*UCSD’s first-year mentorship program helped me find my footing and introduced me to resources, but its usefulness was limited. By my second year, when I needed guidance most, I was left navigating changing interests, career decisions, and the hidden curriculum of higher education alone.*

*Meeting EIL was like a breath of fresh air. He validated the struggles of being a first-generation student, helped make the hidden curriculum visible, and offered practical professional and mental health guidance. But more than anything, he offered consistency. When I retreated into my anxious hyperindependent mode, he checked in and reminded me that I did not have to figure everything out alone — and that this was mutual aid, not charity.*

*Through EIL, I found The Garden, a community rooted in care and belonging. The support we received was not bound by a timeline or paycheck. It was the kind of sustained care I did not know I needed, and it changed how I navigated university and how I want to show up for others.”*

In LC’s experience, YPS provided space to question what a future could look like and to ask for help without shame in a culture that treats career certainty as a prerequisite for belonging. Where LC was navigating institutional ambiguity, AM’s account adds the weight of a family inheritance that had already decided what success was supposed to look like:

*“As the child of Bengali immigrants (AM), success was defined by a predetermined set of benchmarks: get all As, attend an elite college, have a career in engineering or medicine or law with a six-figure salary, a family, a white picket fence, and (maybe) someday, finally dabble in other interests that have plagued your imagination since infancy.*

*It was a step up from the previous generation, where the trajectory was simply to go to school, get married, have kids, and live for everyone but yourself, where servitude was conflated with service. But even this ‘ideal life’ did not prioritize my passions, or even deep engagement with the fields my community wanted for me. Speaking up, stepping out of line, especially in a foreign land, was frowned upon.*

*In moments of confusion about my career path, I turned to RC. They brought me into student government, where I could build on my advocacy for youth in San Diego. With RC, and later EIL and LC, I joined a local nonprofit while still pursuing bioengineering. The Garden watched out for my future before I did, helping me align my family’s hopes with my own visions of learning and service. That spirit of sustenance has stayed with me; from coast to coast, opportunity and support never feel far away.”*

RS, LC, and AM each faced a version of the same barrier, including the hidden curriculum and gatekeeping that treats unfamiliarity as deficiency rather than diversity [59,64]. The Garden provided these three mentees with genuine relationships that allowed them to name the hidden curriculum, form a community they could find belonging in, and pursue a version of success they had defined for themselves.

These accounts suggest that it is vital to consider not only what information students require entering higher education, but also who they trust to hear it from and under what conditions. Near‑peer mentors were able to offer translation without the surveillance, evaluation, or time limits that often accompany formal advising, and to hold tensions that official programming may not make room for [7,26]. Rather than treating YPS as an informal supplement to institutional guidance, universities can recognize YPS as a primary site where the hidden curriculum is actively deconstructed, enhancing students’ ability to succeed [8,17].

## Building infrastructure that reflects lived realities

While many of the services universities provide are well-intentioned and relatively effective, YPS remains a critical, often untapped component within the broader mental health ecosystem; one that, if meaningfully resourced, could significantly strengthen institutions’ overall capacity to care for the students they serve.

Across crisis, identity, and belonging, our lived experiences consistently demonstrate that The Garden regularly provided YPS in practice, although unrecognized and unsupported institutionally. The Garden enabled our collective to survive in an ecosystem with critical gaps, providing wraparound support, translating inaccessible processes, and creating spaces where vulnerability felt safe. These practices mirror, and often exceed, the core competencies and practices that formal peer support frameworks describe as best practice [14,72,73] (see Table 1).

**Table 1:** Competency mapping of youth peer support practices in The Garden.

Peer Support Competencies	Relevant Vignettes	Connection to Practice*
Collaboration & Care	EIL, AZ, RC, MD, GI, KR, RS, LC, AM	YPS was provided both 1-on-1 and as a collective effort within The Garden. In many cases, such as EIL, MD, and AM, multiple members of The Garden collaborated to coordinate specific care depending on the person’s needs.
Provides Support	EIL, AZ, RC, MD, GI, KR, RS, LC, AM	Empathy and intentional support were provided in each mentor-mentee relationship. In multiple cases, support was provided as an accessible alternative to receiving support from university resources.
Recovery Planning	EIL, AZ, RC, MD	Planning centered on the student’s own timeline, needs, and goals rather than the institutions. AZ and RC’s disability accommodations and MD’s departure logistics were built around what each mentee needed, in response to the institution’s limited provided timeframe.
Skill Teaching	EIL, AZ, RC, MD, KR, RS, LC, AM	Members of The Garden were taught a number of skills including how to navigate resources, seek accommodating care, and how to trek higher education’s hidden curriculum while staying true to their identity and values.
Communication	EIL, AZ, RC, MD, KR, RS, LC, AM	Peers advocated for students in both informal and institutional settings, and created space for identity exploration and unlearning (e.g., AZ and RC), that students may have not voiced anywhere else.
Personalization	EIL, AZ, RC, MD, GI, KR, RS, LC, AM	Support was paced to each student’s own bandwidth and readiness. In RC and AZ’s cases, their identity exploration was set by them, not the institution. Additionally, support was provided to directly remedy the institutional and cultural barriers many students faced, with care being personalized to enhance accessibility.
Shares Lived Experience of Recovery	EIL, MD, KR, RC, AZ, RS, LC, AM	Lived experiences, especially of navigating systems and cultural influences on recovery, were consistently drawn upon to provide support. Lived expertise was leveraged to build genuine connection, trust, and inspire hope in each interaction.
Teamwork	EIL, AZ, RC, MD, GI, KR, RS, LC, AM	In many cases, no single peer was supported alone. Oftentimes, The Garden coordinated to provide support, preventing burnout and ensuring that peers received the support they needed.
Resource Sharing	EIL, AZ, RC, MD, KR, RS, LC, AM	Peers made direct, concrete connections to community and institutional resources that students like RC, AZ, and MD may have not been able to navigate alone.
Crisis Management**	EIL, AZ, RC, MD	This competency is visible only where an account involves an explicit acute crisis. EIL’s mentees provided sustained emotional presence, practical support, resources, and accompaniment during a period of passive and acute suicidal distress.
Leadership & Advocacy	EIL, RC, MD, KR, AZ, RS, LC, AM	The Garden repeatedly pushed its members toward leadership roles that they had not seen themselves as qualified for. Beyond interpersonal self-advocacy, oftentimes peers within The Garden supported others to engage in community advocacy efforts, enabling many students to transform their pain into purpose.
Growth & Development	KR, RS, LC, AM	This competency is only visible in accounts that directly pertain to professional development, like RS, KR, LC, and AM. Through the support The Garden provided, each of these mentees were able to enhance their academic experience and genuinely invest in their professional development.

*Competencies were synthesized from the peer support literature, drawing primarily on Hoagwood et al. (2025), Walker et al. (2024), and Davidson et al. (2012). These frameworks were adapted to reflect the organic, non-credentialed context of The Garden, rather than drawn from a single competency taxonomy. Vignettes were written and finalized prior to competency mapping and authors were not prompted using competencies; EIL performed the initial mapping and all co-authors reviewed and confirmed the competencies attributed to their vignettes. This mapping is intended as illustration rather than an independent empirical verification. Where The Garden diverged from professionalized YPS standards (i.e., crisis support) is noted within the relevant cells.**Please note that trained youth peer supporters may engage in crisis response, de-escalation, and resource connection, as described in EIL’s account. However, The Garden diverges from conventional YPS standards, as crisis response is typically accompanied by supervision, formal training, and structured escalation to support youth peer supporters and ensure appropriate triage

Whether academic institutions will recognize it or not, students are innovating their own ecosystems of care, deploying YPS in real time [17]. Whether and how organic, student-led YPS networks form across fraternal, affinity, and cultural organizations in higher education remains an open empirical question the field should prioritize. By recognizing this, institutions can align their infrastructure to enable students to safely and sustainably continue what they are already doing. In order to build a mental health ecosystem that reflects lived realities, universities must meaningfully invest in YPS infrastructure that directly addresses the many reasons students avoid institutionally-based care; centers an intersectional and holistic approach to support; and navigates the critical nuances in trust between marginalized communities and institutions.

The Garden’s collective accounts reflect what organic YPS made possible, but candidly not the extent to which it may burden students. YPS without proper training, supervision, and institutional resources can be personally and psychologically costly, with research on peer supporter burnout documenting the emotional weight of supporting others, even for trained specialists [74,75]. In several instances, co-authors supported others through acute mental health crises while simultaneously navigating their own. The Garden’s lack of an escalation pathway, supervision structure, and institutional acknowledgement ensured the burden fell entirely on the YPWLE providing YPS. We raise this not to undermine the case for investing in student-led YPS, but to clarify precisely the need for investment to include training, supervision, clear escalation protocols, and ongoing support for youth peer supporters themselves. We also note that institutional liability is not generated by funding student-led YPS, it already exists; Students are providing this support without training or institutional knowledge. Investment in training, supervision, and escalation pathways reduce liability rather than introduces it.

Many universities now promote and proclaim “centering student voices” and “shared governance” in their policies and strategies [53,76]. In practice, students often participate in tokenistic, advisory capacities with little to no decision making power [77]. While advisory bodies can be a meaningful gateway for early advocates to influence student health policy, they also risk enacting participatory assimilation [77,78]. UCSD’s own history offers a direct example: the resource centers won through student organizing after Black Winter remain in place today, but the organizers who fought for them have seen their function narrow into routine service delivery, disconnected from the coalition politics that created them [49]. In doing so, the mechanisms advertised as intended to include student voices become an additional layer of gatekeeping that only permits students who are compliant with the university’s vision to lead [77,78].

Meaningful youth leadership mandates youth ownership. The Garden and similar student-run ecosystems of care demonstrate what this can look like in practice. As a community, we self-defined crisis and support, bearing the ethical weight of those choices without training, compensation, or institutional protection. A meaningful YPS infrastructure would not simply absorb this work under strict institutional control, but create conditions in which YPWLE lead program design, governance, and evaluation, with adults in clearly defined supportive roles. By building this infrastructure intentionally, higher education is able to relieve YPS providers of the many barriers cited as reasons for leaving their roles, including burnout, financial sustainability, and organizational culture [74,75].

To ensure the transition from de facto to intentional YPS is authentic, universities must realign their systems across five domains: policy, funding, programming, campus climate, and research (see Fig 1). Together, these shifts would formalize protections and resources for student-run YPS while preserving the autonomy and trust that make it usable to those most marginalized.

**Fig 1:**
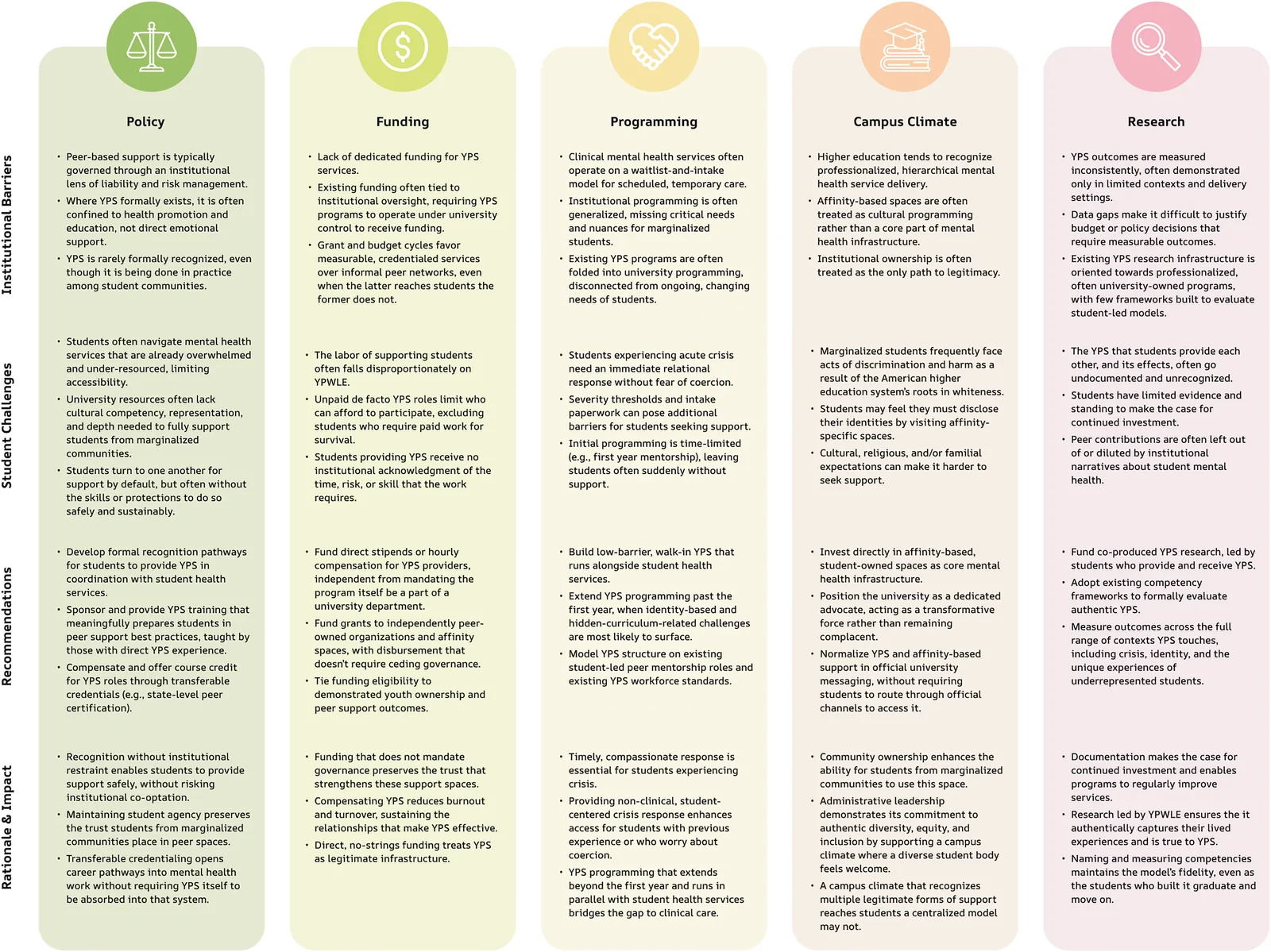
Recommendations for building YPS infrastructure that reflects lived realities. Recommendations organized across five institutional domains (policy, funding, programming, campus climate, and research) that identifies current barriers, student challenges, actionable recommendations, and anticipated impacts across each domain. Icons created by Monkik, bqlqn, Magnific, amonrat rungreangfangsai, and ibrandify from Flaticon (flaticon.com).

Existing YPS trainings, programs, and research may serve as an example of what meaningful, authentic YPS can look like. Youth MOVE National’s Peer Connect program, and Intentional Peer Support both provide a competency taxonomy framework grounded in best practices that are both evidence-based and grounded in the peer movement’s principles and values [79,80]. Independently-governed peer organizations, such as aves Mental Health and The Support Network, illustrate how lived experience and youth-led programs can operate at scale while remaining community governed [81,82]. At the policy level, California’s Peer-to-Peer Youth Mental Health High School Pilot shows that formal, state-funded YPS is achievable even in primary and secondary school settings, where risk management and liability concerns are typically far stricter than in higher education [83]. These examples serve as models that enable universities and student organizations to leverage them and adapt for their own student bodies. The Garden reflects what is already happening among universities, with student mentors, leaders, and organizations across affinity, professional, and social communities facilitating YPS. Effective and responsible scaling of this work requires access to authentic, evidence-based YPS training that equips students with the skills to deliver YPS intentionally, rather than relying on instinct alone

While most members no longer live in close proximity to one another, The Garden has evolved beyond graduation, with connection and support maintained across digital spaces and time zones. To our knowledge, no formal attempt was made to institutionalize The Garden, or similar student-led support systems, after our time at UCSD. The fact that our collective YPS has been sustained, informally, transnationally, and without institutional resourcing, is itself evidence that meaningful peer relationships generate durability that fixed institutional programs may rarely replicate.

The Garden began as a mentor-mentee program and grew into a system of care that held nine of us through crisis. UCSD, and higher education as a whole, are not credited for the creation of that system, but they have always benefited from it. It is unsustainable and unjust to continue relying on invisible, unpaid student labor. Universities have an obligation to recognize, fund, and protect the YPS that is already keeping its most vulnerable students alive.

## Supporting information

S1 Text Author Positionality Statements: Individual positionality statements written by each author, describing their identities, lived experiences, and relationship to the work presented in this essay. (PDF)
