# Supplementary material for: Tending The Garden: What organic youth peer support can teach us about building systems that care
Source: PLOS Ment Health. 2026 Oct 21;3(10):e0000678. doi: 10.1371/journal.pmen.0000678 (PMC13686492; doi:10.1371/journal.pmen.0000678)
Supplement: Supplementary file 1 [file pmen.0000678.s001.pdf]

Supporting Information

S1 Text. Author Positionality Statements

Ernesto Isaac Lara: I am a queer, first-generation college graduate, the grandchild of Mexican immigrants, and a person with living experience of complex trauma. The intersection of these identities has provided me with privilege and marginalization, as well as shaped my experiences navigating higher education and the broader mental health ecosystem. Recognizing the privileges I hold embedded in my position as an American-born citizen with institutional access, I have been able to move through and work within systems in many ways others in my community cannot. Having accessed psychiatric care, counseling, and community-based models of support, youth peer support has championed my recovery, both as a supporter and as the person being supported.

This work is rooted in what I learned as “Broccoli Boy” (the nickname my mentees gave me) in a mentor-mentee community that embodied mutual aid and collective recovery. Becoming “Broccoli Boy” has shaped my early career and grounded my commitment to justice-oriented, liberatory models of care that center lived expertise and collective healing. As a global mental health researcher working at the intersection of advocacy, systems change, and policy reform, I aimed to co-write this essay to challenge the paternalistic and institutional assumption that young people supporting one another is a “liability,” and to name the infrastructure they deserve for the work they are already doing.

Rhianen Callahan: I am a queer transmasc first-generation college graduate of UC San Diego from the San Joaquin Central Valley and a person with lived experience of complex-PTSD. Growing up in a small farm town taught me early on the importance of community when access to resources is limited.

Leana Cortez: I am a first-generation college graduate, researcher of Puerto Rican descent, and a person with living experience of complex trauma. The intersection of these identities molded my experience with peer support and the necessity of it as I navigate systems of higher education that were not designed for folks like me. Experiencing the support of The Garden and similar peer-led systems informs and leads the work and research I do related to community-led health and healing, policy reform, and systems navigation.

Kharylle Rosario: I am a Filipino immigrant and a graduate of UC San Diego. My identities have shaped my experiences in higher education and my approach to interacting with my local communities. Having met my peers through the mentor-mentee community at UC San Diego has played a pivotal role in my growth as a person and as a researcher. My mentor and peers have empowered me throughout my educational and career journey in public health by providing a safe space and equipping me with vital skills in self-advocacy and empathy that have taught me resilience. Whilst I am a student pursuing my MSc in Public Health in London, I often reflect on my time in the mentor-mentee program. Learning to support, provide space for, and empower my

peers has been a generational process. I move forward from this program with the confidence to apply my community-building skills to foster inclusivity and create a welcoming environment within my local communities.

Rocio Sanchez: I am first generation college graduate, first generation Salvadoran American, a Bone Marrow Transplant (HCT) survivor with lived experiences of complex mental health challenges. Navigating through university, imposter syndrome was consuming me and I realized my intersection of identities was not measuring up to my peers. I was in a reoccurring spiral of conflicting emotions but I did not feel I was going through enough hard time to use campus resources. Therefore, mentorships became the forefront of quick contact for discussions and answering questions. With the support of my mentors and The Garden, I have been able to seek resources and transition back to “normal life” following my recovery from my transplant.

Anna Zick: I am a queer woman, a graduate of UC San Diego and San Diego State University, and a person with lived experience of complex-PTSD. Growing up in a conservative, Christian household, I was homeschooled throughout kindergarten to twelfth grade. As the daughter of a military officer, I spent much of my childhood moving across states and countries, rarely staying in one place long enough to build a consistent community and a lasting feeling of belonging. These experiences led me to seek out and explore different campus run clubs and organizations and eventually led me toward the organic, student-run, mentor-mentee communities I found in undergrad. As I graduated undergrad, entered purposeful therapy, and began pursuing my teaching career, the youth peer support I had both provided and received became foundational to how I approach my own education practice. Rooted in my own experiences with The Garden, I strive to create environments of support in which teenagers and young adults can establish their own communities of care.

Aishwarya Mitra: I am a queer, Bengali-Indian bioengineer navigating the push and pull between abiding by the model minority myth and pushing back against destructive systems. The peer support-centered relationships that I was endowed with at UCSD helped me first find my voice and overcome my anxiety. But eventually, their continued presence from wherever I went from coast to coast helped me assess risk when leading direct actions and following the mass shooting at Brown University from where I am on medical leave from my PhD. In The Garden, I am a mentee. But through the peer support network that The Garden has provided, I have learned to emulate the skills in both technical relationships and community building. Our diverse experiences and methods of management have shown me how to assess my safety and the safety of my mentees in solidarity encampments. They taught me to voice my own needs as a quiet person usually scared to disrupt anyone else's life in any way. But I have also learned how to navigate mentees in crisis when we were at a conference in Paris and their professors were unresponsive. Systems like The Garden sustain the youth collective economically, psychologically, and socially. It provides couches to surf on in every city if home ever disappears. It provides a found family to parse through one’s identity. But it also provides a

concrete structure that does take energy from the people inside of it to sustain it. Maybe power-brokers on the outside can help.

Megha Datta: I am a third-culture kid who grew up in Hong Kong, an international student graduate from UC San Diego, and an individual who navigates multiple mental health challenges. While I recognize the immense privilege of having access to an international education, I also know the profound alienation that comes with moving across the world. All at once, I was dealing with massive cultural differences far from my support network, facing the university's hidden curriculum, and realizing I didn’t fit into any of the campus cultural groups. My experience became even more isolating when I survived interpersonal violence during my time on campus. In bouncing between formal psychiatric care and community-based organizations, I found that youth peer support became the bedrock of my survival and recovery. This work is deeply rooted in my lived experience with The Garden, a community of fellow peers that caught me and quietly supported me through daily life when formal institutions weren't entirely equipped to handle the overlapping, nuanced crises that young people face.

Gabriella Imai: I am a first-generation college graduate student who earned her Associate of Arts degree from El Camino Community College and her Bachelor’s degree in Sociology from the University of California, San Diego. As the child of an immigrant mother from Guatemala and a father of Japanese descent, I held high expectations for myself, knowing the immense privilege and positionality I had to challenge myself and pursue opportunities that were not available to my parents. With this in mind, it was difficult for me to navigate the emotions I was experiencing and the challenges I was facing, knowing what a privilege it was to be where I was. My experience became even more difficult to navigate during my final year at the university, and I would not have been able to feel such strong support without the community of peers I met there. They helped me cope with loss and better understand my mental health needs during a significant transitional period in my life. I am deeply grateful to my community and the bond we built together. I attribute the way I move forward in my professional career to their support, guidance and care.
